# Prevalence and Correlates of Self-Harm in the German General Population

**DOI:** 10.1371/journal.pone.0157928

**Published:** 2016-06-30

**Authors:** Astrid Müller, Laurence Claes, Dirk Smits, Elmar Brähler, Martina de Zwaan

**Affiliations:** 1 Department of Psychosomatic Medicine and Psychotherapy, Hannover Medical School, Hannover, Germany; 2 Faculty of Psychology and Educational Sciences, University of Leuven, Leuven, Belgium; 3 Faculty of Medicine and Health Sciences, University Antwerp, Antwerp, Belgium; 4 Odisee University College, Brussels, Belgium; 5 Department of Medical Psychology and Medical Sociology, University of Leipzig, Leipzig, Germany; Central Institute of Mental Health, GERMANY

## Abstract

The study aimed at evaluating the psychometric properties of the German version of the Self- Harm Inventory (SHI) and examining the lifetime prevalence and correlates of self-harm in a representative German population sample (*N* = 2,507; age *mean* = 48.79, *SD* = 18.11; *range* 14 to 94 years; 55.5% women) using the SHI. All participants answered the German SHI, the short form of the Barratt Impulsiveness Scale (BIS-15), the ultra-brief Patient Health Questionnaire for Depression and Anxiety (PHQ-4), and provided sociodemographic information. The one-factorial structure of the SHI was replicated using a confirmatory factor analysis. Internal consistency coefficients were sufficient and in line with previous studies. Almost half of the sample (49%) acknowledged at least one self-harming behavior over the life-span, most frequently indirect forms of self-harm. The rate of participants who engaged in at least one SHI behavior was higher among men than women (51.6% vs. 46.9%, respectively, *χ*^*2*^ *=* 5.38, *p* = 0.020). Higher SHI scores were related to younger age, male gender, living alone, more symptoms of anxiety and depression (PHQ-4), higher impulsivity scores (BIS-15), and suffering from obesity grade 2. Women engaged more often in discreet forms of self-harm than men, e.g., preventing wounds from healing, exercising an injury, starving, and abusing laxatives. In terms of other indirect self-harming behaviors, men admitted more often driving recklessly, being promiscuous and losing a job on purpose, while women reported more frequently engaging in emotionally abusive relationships. With respect to direct self-harm, women were more likely to endorse suicide attempts and cutting, while men admitted more often head-banging. The findings suggest that self-harm constitutes a common problem. Future longitudinal studies are required to examine the natural course, sociodemographic and psychopathological risk factors, as well as possible time-trends of self-harming behaviors in more depth.

## Introduction

Self-harm is conceptualized as a wide range of self-directed harmful behaviors, regardless of their suicidal intent. According to Sansone, Wiederman, and Sansone [1], it “*probably exists along a continuum from graphic*, *self-harm behavior to milder forms of self-sabotaging behavior that might be viewed as self-defeating”* (p. 973). This means that the broad category of deliberate self-harm includes both, direct as well as indirect forms of self-damaging behaviors. It is noteworthy that terms such as “self-harm” and “self-injury” are often used synonymously in the literature, which can be a source of confusion [2, 3]. Self-injury refers to socially unaccepted direct and deliberate destruction (i.e. direct self-harm) of one’s own body surface (e.g. cutting, burning, scratching, biting) without suicidal intent and does not involve indirect forms of self-damaging behaviors [2, 4, 5]. Indirect self-harm is conceptualized as behavior that is also damaging to the self but does not encompass deliberate damage to body tissue. Typical indirect self-harming behaviors are engagement in risky and recklessness behaviors or abusive relationships, disordered eating, substance abuse, etc. [6]. Another discrepancy in the literature pertains to the inclusion vs. exclusion of suicidal intention in the definition of self-harm. Researchers from the UK define self-harming behaviors regardless of suicidal intention, whereas in the U.S.A. self-injury is considered as self-damaging behavior without suicidal intent [7]. Non-suicidal self-injury (NSSI) has been included as a separate clinical syndrome in Section III of the 5^th^ edition of the Diagnostic and Statistical Manual of Mental Disorders (DSM-5) [8]. Although NSSI refers to the direct injury of one’s own bodily tissue without suicidal intent, it can increase the risk for suicide attempts [4, 9]. Accordingly, many authors argue that it is essential to routinely assess the frequency and the functions of NSSI in order to prevent death by suicide [4, 5, 10]. Nock and Prinstein [11] described four primary functions of NSSI that differ along two dichotomous dimensions. The first dimension refers to contingencies that are related to the self (autonomous) or to others (social); the second dimension refers to positive (i.e., the presentation of a favorable stimulus) or negative reinforcement (i.e., the removal of an aversive stimulus). By crossing these two dimensions, four primary function domains appear: automatic-positive reinforcement (e.g., engaging in NSSI to feel pain, which is considered as positive), automatic-negative reinforcement (e.g., engaging in NSSI to remove negative feelings), social-positive reinforcement (e.g., NSSI is followed by attention from others), and finally, social-negative reinforcement (e.g., engaging in NSSI to escape from social demands). Automatic-negative reinforcement is the most frequently reported function of NSSI [11].

Thereinafter, we use the term “self-harm” focusing on the phenomenon in a broader sense, including direct and indirect forms of self-harm with and without suicidal intent. It may occur at all ages, with a peak in adolescence and young adulthood [12]. The large-scale Child and Adolescent Self-harm in Europe (CASE) Study (*n* = 30,477) indicated that 2.6% of the 15 to 16 years old school students had single and 3.2% had multiple episodes of suicidal and non-suicidal self-harm during the past year [13]. Findings from an Australian longitudinal study using a single item question to assess self-harm (“In the last reference period have you ever deliberately hurt yourself or done anything that you knew might have harmed you or even killed you?”) suggested that most self-harming behaviors in adolescents resolve spontaneously to young adulthood [14]. NSSI [15] as well as suicidal and non-suicidal self-harm [16] ooccur in the general population but are especially common in clinical samples. According to the literature, both suicidal and non-suicidal self-harm are associated with high levels of impulsivity, anxiety, and depression [13, 17–20]. Individuals who engage in self-harm may suffer from several mental disorders. The link between NSSI [21] and non-suicidal and suicidal self-harm [7] and the borderline personality disorder, affective disorders and substance use disorders is well established [22]. There exists also evidence for the association between NSSI (i.e. self-injurious behaviors) and eating disorders [23–25]. Furthermore, it appears that patients with extreme obesity seeking bariatric surgery exhibit high levels of suicidal and non-suicidal self-harm [26].

While the majority of previous studies indicated that female gender is predictive, some researchers have reported equal rates of non-suicidal and suicidal self-harm among men and women [16, 27, 28]. Findings from a study investigating non-suicidal and suicidal self-harm in the UK suggest that individuals living alone are more likely to engage in self-harming behaviors than those in a relationship [29]. No link was found between educational level and non-suicidal and suicidal self-harm among individuals who attended emergency departments in Scandinavia [22].

The Self Harm Inventory (SHI) developed by Sansone et al. [1] is a widely used self-report measure that generates information about a broad range of self-harming behaviors over the life span. The behaviors are—according to Latimer, Covic, Cumming, and Tennant [30]—characterized by physical vs. non-physical (i.e., burn self vs. self-defeating thoughts), direct vs. indirect (i.e., cut self vs. starve oneself), and intrapersonal vs. interpersonal (i.e., overdose vs. be promiscuous) self-harm. The pilot version of the SHI consisted of 41 items that were created in accordance with the literature and the clinical experience of the authors and their teams [1]. In developing the final version of the SHI, items were selected based on their correlation with the Diagnostic Interview for Borderlines (DIB; [31]. Items which did not correlate with the DIB were deleted, leading to the final SHI with 22 items. Later on, a SHI cut-off score of 5 [1] or 11 [30] was suggested to be indicative of borderline personality disorder. However, there is some discussion whether it is appropriate to assess the borderline personality disorder solely based on self-harming behaviors given that this is only of the nine diagnostic criteria of the borderline personality disorder listed in DSM-5 [8].

The items of the SHI were preceded by the following statement “Have you ever intentionally, or on purpose … (e.g.) engaged in cutting yourself?”. The questions ask for lifetime history of engagement in self-harming behaviors and are answered on a Yes / No format; the total score is determined by the total number of endorsed items [1]. In line with the UK definition of self-harming behaviors, the SHI does not differentiate between self-harming behaviors with and without suicidal intent.

The factor structure of the SHI inventory was empirically investigated by Latimer et al. [30], who found support for a one-factor structure of the SHI. Sansone, Songer, and Sellbom [32] rationally derived six symptom clusters, which were however never empirically validated. They referred to a suicidal cluster (e.g., overdosed, attempted suicide), a self-injury cluster (e.g., cut, burned, hit, scratched self, banged head), a substance abuse cluster (e.g., abuse prescribed medication, laxatives, alcohol), a cluster referring to abusive relationships (e.g., engage in emotionally abusive/sexually abusive relationships) and a cluster of medically self-defeating behaviors (e.g., prevent wounds from healing, exercised an injury on purpose).

The reliability or internal consistency of the total SHI was investigated in different samples and proved to be very good, with alpha coefficients ranging from 0.80 to 0.90; 0.80 in a sample of 107 psychiatric inpatients (57% female; 18–65 years; [32]), 0.83 in a sample of 423 Australian university students 81% females, 17–30 years; [30], 0.89 in a sample of 52 women seeking treatment in an internal medicine clinic (24–70 years; [33]) and, finally, 0.90 in a sample of 94 internal medicine outpatients (60.6% females, 18–65 years; [34].

Latimer et al. [30] investigated the association between the SHI total score and gender and age in a sample of 423 university students (81% females). Females reported significantly more self-harming behaviors than males; whereas younger students (17 to 19 years) reported significantly less self-harm behaviors compared to older students (20 to 30 years) [30].

In addition, Latimer et al. [30] studied the link between the SHI and depression, anxiety and stress. They divided their student sample in three groups based on the SHI total score: low (1 to 4), medium (5 to 10) and high (11+). The results showed significant differences between the three groups concerning depression, anxiety and stress; with increasing levels of depression, anxiety and stress from the low SHI group, over the medium, to the high SHI group [30]. Moreover, several studies have investigated the association between the SHI total score and different measures of borderline personality disorder [1, 35].

While many studies assessed self-harm (most often NSSI) in German adolescents [3, 36, 37], no study to date examined the prevalence and correlates of self-harm (including both direct and indirect self-harming behaviors) in a representative sample of German adults, including older age groups. To the best of our knowledge, the German translation of the SHI was never validated. To fill up this gap, the aims of the present study were twofold. The study was designed to 1) evaluate the psychometric properties of the German version of the SHI, and 2) examine sociodemographic and psychopathological correlates of self-harm in a large-scale population-based adult German sample.

Based on existing studies [1, 30] we hypothesised that the German version of the SHI would be a one-dimensional instrument with good internal consistency. With respect to sociodemographic correlates, we expected higher rates of self-harm among women, no link between self-harm and educational level, and less self-harm among individuals being single compared to those living with a partner. In terms of associated psychopathology we hypothesized to find strong positive correlations between the occurrence of self-harming behaviors and symptoms of anxiety and depression, and impulsivity. Given that previous research reported high prevalence rates of self-harm in extremely obese individuals [26] we presumed a positive association between self-harm and higher weight.

## Procedure

### Ethics Statement

The survey met the ethical guidelines of the International Code of Marketing and Social Research Practice by the International Chamber of Commerce and the European Society for Opinion and Marketing Research. The study was approved by the ethics committee of the University of Leipzig.

### Data Sampling

Data were collected between March and May 2015. A random sample of the German general population older than 14 years of age was selected with the assistance of a demographic consulting company (USUMA, Berlin, Germany). The sampling procedure followed the established guidelines on how to construct a random population sample in Germany when no access to a population roster is possible. This sampling design involves three consecutive steps: in the first step, a grid of 258 regional sampling areas was randomly selected from a roster of such non-overlapping grids that have been centrally assembled to enhance representativeness in stratified regional sampling in Germany. In the second step, a random procedure to select households of the respective area was implemented within all sampling areas. In the final step, one member of the selected household fulfilling the inclusion criteria (age 14 or older, able to read and understand the German language) was sampled randomly in a pre-specified standardized manner. The sampling procedure is designed to yield random samples representative in terms of age, gender, and education of the German population. A first attempt was made for 4,844 addresses. If not at home, a maximum of three attempts was made to contact the selected person. All subjects were visited by a study assistant who informed them about the investigation, obtained written informed consent, and presented them with the questionnaire.

A total sample of 2,576 individuals agreed to participate, of which 63 individuals did not provide information resulting in a sample of 2,513 individuals (51.9% of the addressed individuals). Of those, 130 had missing data on one or more SHI items. There were six cases with missing values on all SHI items. Those cases were removed from the sample leading to a final sample of 2,507 participants.

### Methods

#### Instruments

All participants provided sociodemographic information and filled-out the self-rating instruments described below. The Body Mass Index (BMI) was calculated based on the self-reported current weight in kilogram divided by the squared length in meters (kg/m^2^).

We used a German translation of the SHI that was kindly provided by Dr. Paul Plener from the Department of Child and Adolescent Psychiatry and Psychotherapy, University of Ulm, Germany. The translation of the questionnaire was authorized by the authors of the original version and followed the guidelines for the process of translation of self-reports, including the use of back-translation.

Impulsiveness was measured using the short form of the Barratt Impulsiveness Scale [38] (German version: BIS-15 [39]). The questionnaire contains 15 items that are answered on a 4-point scale ranging from 1 = rarely/never to 4 = almost always/always. Cronbach’s *α* of the BIS-15 in the present sample was 0.83, which is very similar to the study of Meule, Vögele and Kübler [39] who reported a Cronbach’s *α* of 0.81 in their validation study.

The ultra-brief Patient Health Questionnaire for Depression and Anxiety (PHQ-4) [40] was used to assess core symptoms of depression and anxiety disorder. The questionnaire consists of four items that assess the frequency (ranging from 0 = not at all to 3 = nearly every day) of feeling depressed, loss of interest in doing things, feeling nervous/anxious/on edge and not being able to stop or control worrying over the past two weeks. Cronbach’s *α* of the PHQ-4 in the present sample was 0.84.

#### Statistical Analyses

Statistical analyses were performed using Mplus 7.3 and IBM SPSS Statistics version 23, as appropriate. As mentioned above, six cases with missing values on all SHI items were deleted. The remaining dataset (*N* = 2,507) was used without imputation. The full information maximum likelihood (FIML) method was used to handle missing data in order to estimate the model parameters and standard errors in all remaining cases (including those with incomplete SHI data). This method is superior to other methods like pairwise or listwise deletion [41].

Given that previous studies had confirmed a one-factorial structure for the SHI and in order to test the original structure, the one-factor model was tested in the present sample using confirmatory factor analysis (CFA). Second, it was investigated whether this structure generalizes to both gender subsamples. As the SHI responses are dichotomous (Yes or No), a weighted least squares means and variances adjusted estimation method (WLSMV option, MPLUS 7.3) was used for estimating all model parameters. Model fit was assessed by multiple criteria: comparative fit index (CFI) for fit relative to a null model, complemented with the root mean squared error of approximation (RMSEA) for overall fit. The criteria for good model fit were defined according to Hu and Bentler [42] as CFI > 0.95 (0.90 is acceptable), and RMSEA > 0.06 (0.09 is acceptable). The final solution for both gender subsamples was compared to the CFA solution of the total sample using factor congruence coefficients.

Previous research demonstrated a strong relationship between the SHI and measures of impulsivity, depression, and anxiety. Therefore, construct validity was determined calculating correlations between the SHI and the BIS-15 (impulsivity) and the PHQ-4 (depression/anxiety). Because the SHI scores were not normally distributed (i.e. significant Kolmogorov-Smirnov test, significant Shapiro-Wilk test) we used two-tailed Spearman rank correlations.

Weight status was classified using the World Health Organization categories of underweight (BMI < 18.5 kg/m^2^), normal weight (BMI 18.50 to 24.99 kg/m^2^), overweight (BMI 25 to 29.99 kg/m^2^) and obesity grade 1 (BMI 30 to 34.99 kg/m2), grade 2 (BMI 35 to 39.99 kg/m2), and grade 3 (BMI ≥ 40 kg/m2).

Because of the skewed distribution of the SHI total scores we reported the median and the inter-quartile range (IQR) of the SHI total scores besides the mean and standard deviation. In line with research that indicates differences in the occurrence of self-harming behaviors in function of gender, age, years of education, marital status, and weight status, differences in SHI scores in function of these variables were examined using nonparametric tests (i.e. Kruskal-Wallis test, Mann-Whitney *U* tests). Categorical variables were compared by means of the *χ2*-test statistic. The significance level for all tests was set at *α* = 0.05.

## Results

### Description of the sample

The study included 2,507 individuals (1,115 men and 1,392 women) between 14 and 94 years that were divided into seven age groups. Table 1 displays sociodemographic characteristics of the total sample and separately for men and women. There were no significant gender differences with respect to age, years of education, and nationality. Women were more often living without a partner than men. Men had more often a BMI of ≥ 25 kg/m^2^ than women (61.1% vs. 50.3%, respectively, *χ*^*2*^ *=* 29.10, *p* < 0.001).

**Table 1:** Sample characteristics.

	Total sample		Men	Women	Comparison men vs. women
	*N*	mean *(SD)*	mean *(SD)*	mean *(SD)*	
Age [years]	2507	48.79 (18.11)	48.31 (18.17)	49.17 (18.05)	*t* = 1.18
		*n* (%)	*n* (%)	*n* (%)	
Age Groups [years]	2507				*χ*^*2*^ *=* 7.68
≤ 24		276 (11.0)	139 (12.5)	137 (9.8)	
25 to 34		376 (15.0)	159 (14.3)	217 (15.6)	
35 to 44		374 (14.9)	166 (14.9)	208 (14.9)	
45 to 54		469 (18.7)	196 (17.6)	273 (19.6)	
55 to 64		460 (18.3)	215 (19.3)	245 (17.6)	
65 to 74		347 (13.8)	155 (13.9)	192 (13.8)	
≥ 75		205 (8.2)	85 (7.6)	120 (8.6)	
Marital status	2498				*χ*^*2*^ *=* 11.29**
Married/living together		1102 (44.1)	532 (47.8)	570 (41.1)	
Living apart/single/divorced/widowed		1396 (55.9)	580 (52.2)	816 (58.9)	
Nationality	2507				*χ*^*2*^ < 0.01
German		2421 (96.6)	1077 (96.6)	1344 (96.6)	
Other		86 (3.4)	38 (3.4)	48 (3.4)	
School years	2507				*χ*^*2*^ = 0.81
≥ 12 years		517 (20.6)	239 (21.4)	278 (20.0)	
< 12 years		1990 (79.4)	876 (78.6)	1114 (80.0)	
Weight categories	2465				*χ*^*2*^ *=* 66.10***
underweight		40 (1.6)	7 (0.6)	33 (2.4)	
normalweight		1071 (42.6)	419 (38.2)	648 (47.3)	
overweight		967 (38.5)	512 (46.7)	455 (33.2)	
obesity grade 1		246 (9.8)	115 (10.5)	129 (9.4)	
obesity grade 2		102 (4.1)	30 (2.7)	72 (5.3)	

Note.

** *p* < 0.01,

*** *p* < 0.01

### Factor Structure, reliability, and construct validity of the German SHI

The results of the CFA are listed in Table 2. According to the cut-off values for the fit indices, the one-factor model showed a reasonable fit in the total sample. Subsequently, the sample was split into a male (*n* = 1,115) and a female subgroup (*n* = 1,392). In both subsamples, a one-factor model was fitted by means of CFA. For the male sample, items 21 and 22 had to be removed because they were endorsed by less than 0.04% of the sample, resulting in insufficient variation in the responses to allow for a reliable estimation of the parameters associated with those items. For the female sample, all items could be included as in the total sample. The results of the CFA indicated an acceptable fit for the one-factor model for both, the male and the female sample separately (Table 2).

**Table 2:** Fit indices of the confirmatory factor analysis for the Self Harm Inventory.

	*N*	*Chi^2^*	*df*	*CFI*	*RMSEA*
Total sample	2507	758.57	209	0.93	0.04
Men^a^	1115	287.84	170	0.97	0.03
Women	1392	477.36	209	0.94	0.03

Note.

^a^ Items 21 and 22 had to be removed because they were endorsed by less than 0.04% of male participants.

df = degree of freedom, CFI = Comparative Fit Index, RMSEA = Root Mean Square Standard Error of Approximation.

Factor congruence coefficients for the one-factor solution for females and for males versus the total sample were both equal to 0.99, meaning that the pattern of factor loadings was very similar in the total sample and both gender groups. Table 3 displays the factor loadings for the one-factor solution in the total sample as well as in the male and female participants. Factor loadings of item 17 were substantially lower than loadings of any other item, though the endorsement was high. The total SHI showed acceptable internal consistency (reliability) with Cronbach’s *α* coefficients of 0.78 for the total sample, 0.75 for men (same result if items 21 and 22 were excluded), and 0.80 for women.

**Table 3:** Factor loadings for the 1 factor solution of the total sample and of the male and female participants separately.

Item	Total sample	Male Sample^a^	Female Sample
1	0.79	0.85	0.76
2	0.91	0.94	0.91
3	0.90	0.95	0.90
4	0.77	0.80	0.78
5	0.71	0.72	0.74
6	0.78	0.84	0.75
7	0.69	0.75	0.64
8	0.77	0.65	0.83
9	0.74	0.75	0.75
10	0.67	0.65	0.69
11	0.64	0.69	0.62
12	0.71	0.69	0.73
13	0.79	0.81	0.77
14	0.67	0.64	0.70
15	0.72	0.75	0.72
16	0.79	0.78	0.80
17	0.39	0.43	0.37
18	0.78	0.73	0.81
19	0.89	0.85	0.91
20	0.70	0.66	0.75
21	0.72	-	0.78
22	0.65	-	0.70

Note.

^a^ Items 21 and 22 had to be removed because they were endorsed by less than 0.04% of male participants.

Table 4 displays the results of the Spearman correlations between the SHI and the BIS-15, and the PHQ-4 in the total sample, and separately for men and women. As expected, higher scores on the SHI were moderately related to more impulsiveness (BIS-15) and more symptoms of anxiety and depression (PHQ-4).

**Table 4:** Two-tailed Spearman rank correlations between the Self Harm Inventory (SHI), the 15-item Barratt Impulsiveness Scale (BIS-15), and the ultra-brief Patient Health Questionnaire for Depression and Anxiety (PHQ-4)

	Total sample*N* = 2,292	Men*n* = 1,032	Women*n* = 1,260
	SHI		
BIS-15	0.23**	0.26**	0.20**
PHQ-4	0.37**	0.36**	0.40**

Note.

Listwise deletion of missing data was used.

** *p* < 0.01

### Prevalence and correlates of self-harming behaviors

Table 5 summarizes the frequencies of self-harming behaviors as measured with the SHI in the total sample and separately for men and women. Overall, 1,228 participants (49.0% of the total sample) endorsed at least one SHI item. The behaviors with the highest prevalence rates were “torturing oneself with self-defeating thoughts” (29.8%), “lost a job on purpose” (24.4%), and “abused alcohol” (13.60%). Seventy-one individuals (2.8%) acknowledged “suicide attempts”.

**Table 5:** Frequencies of self-harming behaviors for the total sample, and separately for men and women.

	Total sample		Men	Women	Comparison men vs.women
	*N*	n (%)	n (%)	n (%)	*χ*^*2*^
1. Overdosed	2501	61 (2.4)	25 (2.2)	36 (2.6)	0.31
2. Cut yourself	2501	61 (2.4)	15 (1.3)	46 (3.3)	10.04**
3. Burned yourself	2502	18 (0.7)	8 (0.7)	10 (0.7)	<0.01
4. Hit yourself	2499	37 (1.5)	20 (1.8)	17 (1.2)	1.40
5. Banged your head	2498	74 (3.0)	51 (4.6)	23 (1.7)	18.58***
6. Abused alcohol	2496	339 (13.6)	212 (19.1)	127 (9.2)	51.84***
7. Driven recklessly	2494	237 (9.5)	159 (14.3)	78 (5.6)	53.87***
8. Scratched yourself	2499	87 (3.5)	32 (2.9)	55 (4.0)	2.13
9. Prevented wounds fromhealing	2501	65 (2.6)	20 (1.8)	45 (3.2)	5.04*
10. Made medical situationsworse	2496	104 (4.2)	44 (4.0)	60 (4.3)	0.20
11. Been promiscuous	2497	125 (5.0)	82 (7.4)	43 (3.1)	23.92***
12. Set yourself up in arelationship to be rejected	2497	66 (2.6)	31 (2.8)	35 (2.5)	0.17
13. Abused prescriptionmedication	2501	73 (2.9)	32 (2.9)	41 (2.9)	0.01
14. Distanced yourself from Godas punishment	2496	34 (1.4)	17 (1.5)	17 (1.2)	0.43
15. Engaged in emotionallyabusive relationships	2496	145 (5.8)	51 (4.6)	94 (6.8)	5.34*
16. Engaged in sexually abusiverelationships	2494	96 (3.8)	39 (3.5)	57 (4.1)	0.57
17. Lost a job on purpose	2501	609 (24.4)	294 (26.4)	315 (22.7)	4.54*
18. Attempted suicide	2500	71 (2.8)	18 (1.6)	53 (3.8)	10.87**
19. Exercised an injury	2498	51 (2.0)	12 (1.1)	39 (2.8)	9.28**
20. Tortured yourself with self-defeating thoughts	2497	744 (29.8)	313 (28.2)	432 (31.1)	2.52
21. Starved yourself to hurtyourself	2499	41 (1.6)	4 (0.4)	37 (2.7)	20.33***^a^
22. Abused laxatives to hurtyourself	2492	14 (0.6)	1 (0.1)	13 (0.9)	7.89**^a^

Note.

*ns* = not significant,

* p < 0.05,

** *p* < 0.01,

*** *p* < 0.01,

^a^ two-tailed Fisher’s exacttest was used due to the low cell count

#### Gender

**Fig 1:**
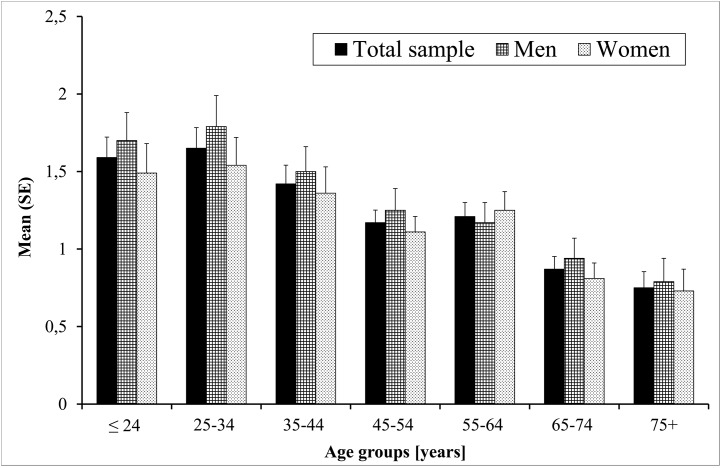
Comparison of Self Harm Inventory scores across age groups in the total sample and separately for men and women.

The rate of participants who engaged in at least one SHI behavior was higher among men than women (51.6% vs. 46.9%, respectively, *χ*^*2*^ *=* 5.38, *p* = 0.020). In the same vein, men exhibited significantly higher SHI total scores than women (*mean*_*m*_ = 1.33, *SD*_*m*_ = 2.03, *median*_*m*_ = 1, *IQR*_*m*_ = 2 vs. *mean*_*w*_ = 1.20, *SD*_*w*_ = 2.05, *median*_*w*_ = 0, *IQR*_*w*_ = 2; *U* = 735777.50, *p* = 0.016). Separate analyses for each of the seven age groups listed in Table 1 partly confirmed the aforementioned gender differences (see Fig 1). Only among participant younger than 24 years (60.4% vs. 49.3%, *χ*^*2*^ *=* 4.53, *p* = 0.033) and those between 25 and 34 years (51.6% vs. 46.9%, *χ*^*2*^ *=* 5.38, *p* = 0.020) the percentage of participants endorsing at least one SHI item was higher in male than in female individuals. With regard to total SHI scores, men and women did not differ significantly across the seven age groups. There was a trend toward higher SHI total scores in men compared to women in participants between 25 and 34 years (*mean*_*m*_ = 1.79, *SD*_*m*_ = 2.50, *median*_*m*_ = 1, *IQR*_*m*_ = 2 vs. *mean*_*w*_ = 1.54, *SD*_*w*_ = 2.64, *median*_*w*_ = 0, *IQR*_*w*_ = 2; *U* = 15345.50, *p* = 0.053).

We also examined whether there was a gender difference in rates of endorsement for each of the 22 SHI items separately. As can be seen in Table 5, more men than women endorsed the items “banged your head”, “abused alcohol”, “lost a job on purpose”, “driven recklessly”, and “been promiscuous”. In contrast, more women than men admitted “cutting”, “preventing wounds from healing”, “engaging in emotionally abusive relationships”, “exercising an injury”, “starving”, and “abusing laxatives”. In addition, more women than men acknowledged “suicide attempts”.

Although the direction of the aforementioned findings did not differ by age groups, there were only two items with consistently significant gender differences, regardless of age. These items were “abused alcohol” and “driven recklessly”, which were more often approved by men than women within all seven age groups (all *p*.s < 0.035). Most gender differences (i.e. five out of 22 SHI items) were found in participants aged between 35 and 44 years. In this age group, more men than women admitted that they had “abused alcohol”, “driven recklessly”, and “lost a job on purpose” (all *p*.s < 0.025), while more women than men indicated “cutting” and “preventing wounds from healing” (all *p*.s < 0.026). “Banged your head” was more often indicated by men aged <24 years, between 45 and 54 years, and those between 65 and 74 years (all *p*.s < 0.040). The item “been promiscuous” was more often affirmed by men between 25 and 34 years, and in those aged between 45 to 54 years (all *p*.s < 0.010).

#### Age

Higher SHI scores were related to lower age in the total sample (*r* = -0.19, *p* < 0.01), as well as in the male (*r* = -0.15, *p* < 0.01) and female (*r* = -0.10, *p* < 0.01) subsamples. 0 1 indicates a decrease in the number of self-harming behaviors from the younger to the older age groups in the total sample (Kruskal-Wallis test: *χ*^*2*^ = 48.52, *df* = 6, *p* < 0.001). The two oldest age groups (years: 75 to 74 and 75+) differed significantly from all other age groups (all *p*.s < 0.01). Separate overall group comparisons for men (*χ*^*2*^ = 31.06, *df* = 6, *p* < 0.001) and women (*χ*^*2*^ = 22.05, *df* = 6, *p* = 0.001) revealed similar results. In the male sample the two oldest age group scored lower on the SHI than all individuals ≤ 54 years (all *p*.s < 0.05), and in the female sample lower than all participants ≤ 64 years (all *p*.s < 0.03).

#### Partnership status and education

Individuals currently living together with a partner scored considerably lower on the SHI (*n* = 1,102; *mean* = 0.91, *SD* = 1.56, *median* = 0, *IRQ* = 1) than those without a partner (i.e. living apart, being single, divorced or widowed; *n* = 1,396; *mean* = 1.53, *SD* = 2.31, *median* = 1, *IQR* = 2; *U* = 646214.50, *p* < 0.001). Subsequent separate analyses for men and women revealed similar differences.

Individuals who had completed 12 or more years of education reported higher SHI scores (*n* = 517; *mean* = 1.49, *SD* = 2.14, median = 1, *IQR* = 2) than those with less years of education (*n* = 1,990; *mean* = 1.20, *SD* = 2.01, *median* = 0, *IQR* = 2; *U* = 470116.50, *p* = 0.001). A comparison between men and women indicated a gender effect. Women with 12 or more years of education exhibited higher SHI levels (*n* = 278; *mean* = 1.47, *SD* = 2.10, *median* = 1, *IQR* = 2) than women reporting less years of education (*n* = 1,114; *mean* = 1.13, *SD* = 2.04, *median* = 0, *IQR* = 1; *U* = 137180.00, *p* = 0.001). In men, no relationship was found between SHI scores and years of education (≥12 years: *mean* = 1.51, *SD* = 2.19, *median* = 1, *IQR* = 2; <12 years: *mean* = 1.28, *SD* = 1.98, *median =* 1, *IQR* = 2; *U* = 99253.00, *p* = 0.188).

#### Weight Status

We also explored if the number of participants who endorsed at least one SHI item was related to weight status. In the total sample, we assessed the following rates of any self-harm within BMI groups: underweight 40%, normal weight 50%, overweight 46.3%, obesity grade 1 52.9%, obesity grade 2 60.8%, and obesity grade 3 57.8% (*χ*^*2*^ = 12.96, *df* = 5, *p* = 0.024). Between-group comparisons revealed significant differences between individuals with obesity grade 2 and those with underweight, normal weight, and overweight (all *p*.*s* ≤ 0.038), in that individuals with grade 2 obesity reported significantly higher levels of self-harm. No significant relationships between SHI and BMI groups were observed for men (*χ*^*2*^ = 7.18, *df* = 5, *p* = 0.208) and women (*χ*^*2*^ = 8.13, *df* = 5, *p* = 0.149) separately.

**Fig 2:**
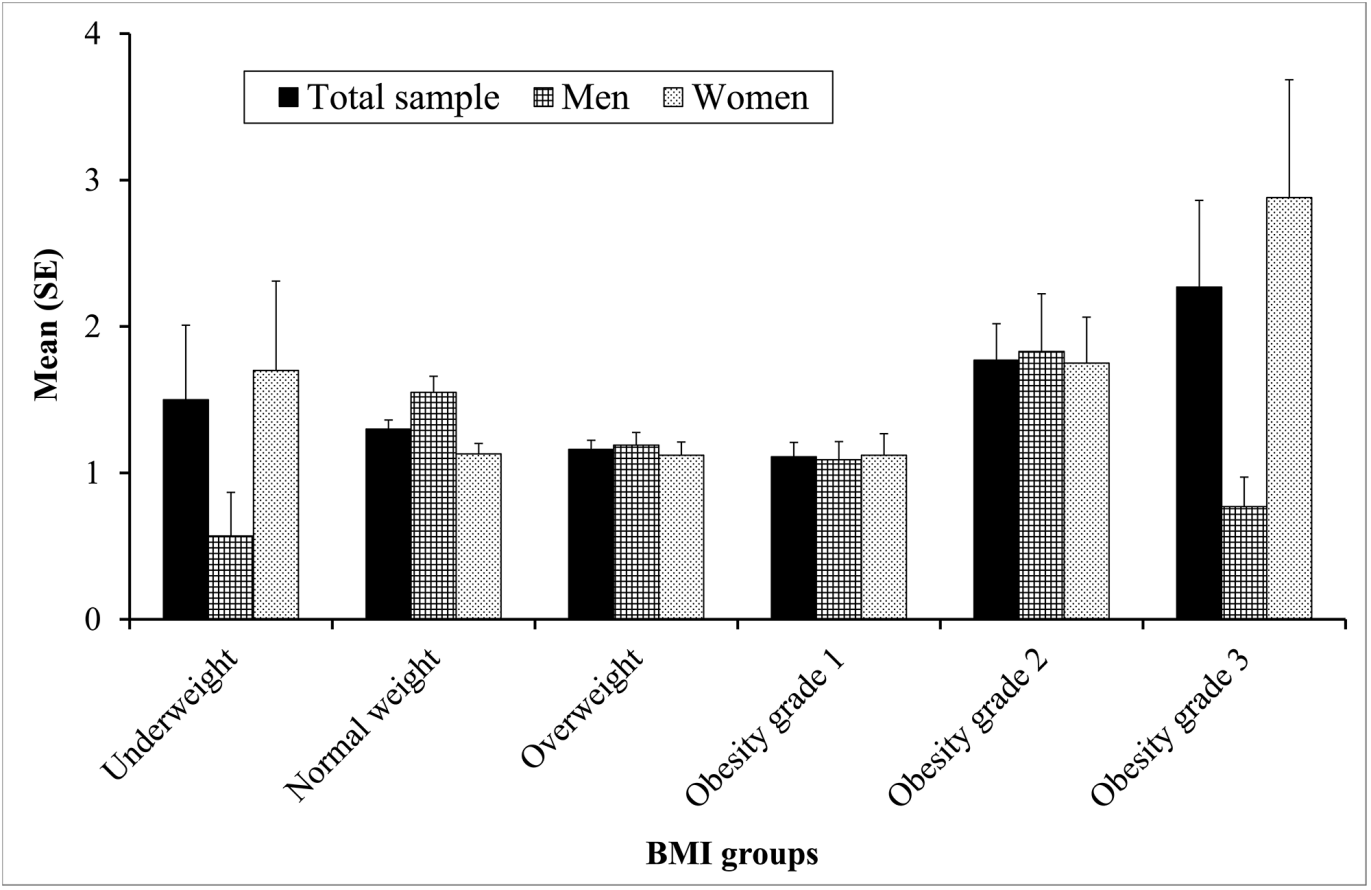
Comparison of Self Harm Inventory scores across BMI groups in the total sample and separately for men and women.

Continuous SHI total scores were not related to BMI scores according to bivariate correlations (*r* = 0.004, *p* = 0.832). Nonetheless, Fig 2 suggests higher SHI scores in obese participants (Kruskal-Wallis test: *χ*^*2*^ = 15.28, *df* = 5, *p* = 0.009). Pairwise comparisons showed that the obesity grade 2 group exhibited higher SHI scores than all groups with a lower BMI (all *p*.s < 0.046). The overweight group had lower SHI scores than the obesity grade 3 group (*p* = 0.046). No differences were found between the other groups. In terms of gender differences, subsequent analyses did not show a significant relationship between BMI groups and SHI scores in the male (*χ*^*2*^ = 9.86, *df* = 5, *p* = 0.079) but in the female sample (*χ*^*2*^ = 11.79, *df* = 5, *p* = 0.038) (see Fig 2). Pairwise group comparisons showed that women from the normal and the overweight group exhibited significantly less self-harm than women with obesity grade 2 or 3 (all *p*.s < 0.032). The remaining groups did not significantly differ from each other.

## Discussion

Below we discuss our findings with regard to the factor structure and reliability of the German SHI, the occurrence of non-suicidal and suicidal self-harm in the present German population sample, and the sociodemographic and psychopathological correlates of self-harm.

### Factor structure and reliability of the German SHI

Overall, the present findings support our hypothesis pertaining to the one-factorial structure of the German SHI. Testing the fit of the single-factor model proposed by Sansone et al. [1] by conducting a CFA, the model provided a reasonably acceptable, although not excellent, fit to the data. Internal consistency coefficients (reliability) of the German version of the SHI for the total sample, males and females were sufficient and in line with previous data [30, 32, 33, 35]. With regard to the low loadings of item 17 (“Lost a job on purpose”), we may assume that this item refers to a form of self-harming behavior which is less related to the direct and/or indirect harm to the body compared to the other SHI items (e.g. like self-cutting, sexual abusive relationships).

### Prevalence and sociodemographic correlates of self-harm

This was the first study that investigated frequencies of self-harming behaviors in the general German population using the SHI. Almost half of the sample acknowledged at least one self-harming behavior, most frequently cognitive (“torturing with self-defeating thoughts”), interpersonal (“losing a job on purpose”), and other forms of indirect (”abused alcohol”) self-harm. This is partially in line with the findings of Latimer et al. [30]. In their study, “torturing with self-defeating thoughts” and “abused alcohol” were the most frequently endorsed items. “Lost a job on purpose” was less often acknowledged, which can be explained by the fact that Latimer et al. investigated university students who might not have had many chances to lose a job.

Past research indicated higher prevalence rates of NSSI behaviors among women compared to men [21, 47]. Though, some researchers reported equal prevalence rates among men and women in non-clinical samples [27]. When considering the total sample, our findings suggest that men are even more likely to engage in NSSI than women. A more detailed analysis by age groups, however, suggested that this was only true for two out of seven age groups, particularly those younger than 34 years. No gender effect was found in older age groups, which is in line with earlier findings [27] and highlights the occurrence of self-harm in both gender groups.

Consistent with past research concerning gender differences in NSSI [43], we found discrepancies between male and female participants in the methods of self-harm. There were gender differences in the frequencies of 12 out of 22 self-harming behaviors in the present sample (see Table 5). With respect to direct self-harm, women were more likely to endorse suicide attempts and cutting, while men admitted more often head-banging. It is noteworthy that cutting has been described as a typically female NSSI method in earlier studies investigating gender differences in psychiatric inpatients [44] and in undergraduate students [45].

In the present sample, women exhibited more often discreet forms of self-harm than men, e.g. preventing wounds from healing, exercising an injury, starving, and abusing laxatives. In terms of indirect self-harming behaviors, men admitted more often driving recklessly, being promiscuous and losing a job on purpose, while women reported more frequently engaging in emotionally abusive relationships. This gender-related pattern of indirect non-suicidal and suicidal self-harm methods is in line with the study of Claes, Vandereycken and Vertommen [44] who found that male compared to female patients admitted more often alcohol abuse, driving recklessly, promiscuity, and losing a job on purpose, while female patients acknowledged more often starvation and laxative abuse than male patients.

The observed negative correlation between age and self-harm raises the question of whether self-harm declines with older ages. It is noteworthy that the SHI does not assess the current but the life-time prevalence of self-harm. Unfortunately, we did not assess the age of onset of self-harming behaviors and the cross-sectional design prevents any causal interpretation of the negative association between age and self-harm frequency. Alternative explanations of the negative correlation between SHI scores and age include reasons such as selective mortality and time trends in self-harm incidence. It is well known that self-harm constitutes a risk factor for suicide [9]. The lower level of self-harm in older age groups may be explained by the death of a significant proportion of individuals suffering from self-harm earlier in life, regardless of the cause of death. With regard to time trends, the underlying assumption is that the occurrence of self-harm may have increased over the last decades resulting in higher prevalence rates among younger age groups. In fact, Hawton et al. [46] referred to rising numbers of non-suicidal and suicidal self-harm patients in Oxford (United Kingdom) between 1985 and 1995, most markedly in young men, and to changes in the characteristics of self-harm patients between 1990 and 2000 [29]. The same research group examined trends in self-harm incidence in Oxford between 1996 and 2010 and described an initial increase of self-harm until 2003 and a subsequent decline [47]. The question of whether aging decreases the occurrence of self-harm requires further testing using a longitudinal study design.

With regard to education, our results pertaining to male participants support the findings of others [22, 47] who did not find an association between non-suicidal and suicidal self-harm and education history. Though, at least in the present sample women with more years of education reported more self-harming behaviors. The latter finding can be connected with findings from a previous study in a representative British community sample [48]. This study suggested an increase in the prevalence of borderline personality disorder symptoms in higher social classes that might imply a link between self-harm and higher educational level [49].

In line with previous studies concerning correlates of borderline personality disorder [50, 51], participants currently living with a partner exhibited less self-harm than those being single, living apart, being divorced, or widowed. This result leads to the question if marital status constitutes a protective factor against self-harm or if self-harm prevents successful partnership. Due to the cross-sectional design and the lack of information about the characteristics and quality of existing partnerships we are not able to draw causal conclusions.

### Self-harm and associated psychopathology

The present study replicated the positive association between the level of non-suicidal and suicidal self-harm and symptoms of anxiety and depression as suggested by many other authors [7, 13, 17–19, 22, 30, 32], and the positive association between the level of non-suicidal and suicidal self-harm and impulsivity [18–20]. These findings confirm the good construct validity of the German SHI. The correlations between the SHI and the PHQ-4 (anxiety/depression), and the BIS-15 (impulsivity) were not strong but moderate, which may be explained by the use of a community sample. One may assume that the correlations would have been stronger in a clinical sample with higher level of general psychopathology.

Another interesting outcome is the link between obesity and increased levels of self-harm, especially among female participants. Sansone, Wiederman, Schurmacher, and Routsong-Weichers [26] investigated non-suicidal and suicidal self-harm in 121 morbidly obese bariatric surgery candidates and found a relatively high rate of self-harm (53.7%) in this population, without gender differences. In the present community sample, we found similarly high rates of self-harm among obese participants ranging from 52.9% to 60.8%. At present, the literature concerning self-harm in morbid obesity is still sparse. The growing research indicating elevated risk of suicide after bariatric surgery [52, 53] and the fact that recurrent self-harm is associated with increased risk for suicide attempts [9] underscores the need for future studies investigating the association between obesity and self-harming behaviors in more detail.

### Strengths and limitations

The replication of the one-factorial structure facilitates the comparison of German SHI data with studies that utilized the original SHI version. Another strength of the present study is the use of a large representative population sample and the availability of sociodemographic data and information about clinical symptoms and weight status. To date, this is the first study that examined a broad range of self-harming behaviors in the adult German population; however, not making a differentiation between the non-suicidal or suicidal intent of the behaviour. The present findings add to an overall body of evidence demonstrating that self-harm is a significant public health problem.

Several limitations are important to note. Although the one-factor structure was consistent with the original SHI, other factor structures were not examined. The cross-sectional design prevents causal conclusions about the relationships between variables. Future longitudinal studies are needed to examine the natural course of self-harm in nonclinical samples and its correlates. Furthermore, we cannot exclude that the rates of self-harming behaviors were distorted by social desirability bias, which is another shortcoming. Additionally, it is possible that the correlations between variables are increased due to shared method variants (all self-reports); so future studies should include interviews or performance-based measures (besides self-report measures) of the variables under study.

## Conclusions

The German version of the SHI represents a brief, useful measure to assess a broad variety of concrete self-harming behaviors in clinical practice as well as in empirical studies. By using this instrument, the rate of those who exhibited at least one self-harming behavior over the life-span was relatively high (49%) in the present representative German sample. This finding suggests that self-harm constitutes a common problem. Future longitudinal studies are required to examine the natural course, sociodemographic and psychopathological risk factors, as well as possible time-trends of self-harming behaviors in more depth.
